# Intrathecal drug delivery to treat intractable neuropathic pain following Sjögren's syndrome-induced transverse myelitis: A case report

**DOI:** 10.1097/MD.0000000000026141

**Published:** 2021-06-04

**Authors:** Ji Yeong Kim, Yong Ho Lee, Ji Young Kim, Hyun Hwa Lee, Young Hoon Kim

**Affiliations:** a Department of Anesthesiology and Pain Medicine, Yonsei University College of Medicine, Gangnam Severance Hospital, Seoul; b Department of Anesthesiology and Pain Medicine, Keimyung University Dongsan Hospital, Daegu; c Department of Anesthesiology and Pain Medicine, Seoul St. Mary's Hospital, College of Medicine, The Catholic University of Korea, Seoul, Republic of Korea.

**Keywords:** intrathecal morphine pump, neuropathic pain, Sjögren's syndrome, transverse myelitis

## Abstract

**Rationale:**

Transverse myelitis (TM) is a spinal cord inflammatory myelopathy that causes motor/sensory loss and urinary retention below the level of the affected spinal cord. Although a few case reports have described the control of neuropathic pain in patients with TM via spinal cord stimulation, no documented case regarding the control of severe allodynia following TM via intrathecal pump has been described.

**Patient concerns:**

A 37-year-old woman was referred to a pain clinic for severe intractable pain below the T5 level followed by Sjögren's syndrome-induced TM.

**Diagnoses:**

A neurological examination revealed paresthesia and allodynia below the T5 level. The sensory evaluation was limited by extreme pain and jerking movements. The muscle strength of both lower limbs was grade 3.

**Interventions:**

Intrathecal pump was inserted into the left lower abdomen. Catheter tip was placed at the midline of the T8 level.

**Outcomes:**

The numeric rating scale (NRS) for pain score decreased from 10 to 5. Functional Independence Measure score increased from 67 before implantation to 92 at the time of discharge, while the patient's Barthel score increased from 31 to 46.

**Lessons:**

Neuropathic pain due to Sjögren's syndrome-related TM could be controlled effectively using the intrathecal morphine pump.

## 1 Introduction

Patients with Sjögren's syndrome have a 20% chance of experiencing neurological disease.^[1]^ Neurological symptoms may be early symptoms of Sjögren's syndrome, preceding sicca symptoms.^[1]^ Sjögren's syndrome can invade both the peripheral nervous system and the central nervous system, with central nervous system being less common but more lethal.^[2]^ Among spinal cord lesions, transverse myelitis (TM) is most likely to coexist with Sjögren's syndrome.^[3]^ TM is a spinal inflammatory myelopathy that causes motor and sensory loss, as well as urinary retention below the level of the affected spinal cord. The incidence of TM in Sjögren's syndrome is 1%.^[4]^ Although it occurs rarely, if neuropathic pain remains as a late complication of TM, physicians may encounter challenging circumstances.^[4]^ Traditional treatments for neuropathic pain include anticonvulsant, antidepressant, sympathetic block, spinal cord stimulation (SCS), and intrathecal morphine pump (ITP).^[5]^ However, the prognosis is poor, and usually intractable chronic courses are followed. A few case reports have described the control of neuropathic pain in patients with TM via SCS.^[6–8]^ Here, we present a successful case of pain control after inserting an ITP into a patient with recurring Sjögren's syndrome-related TM. This study was approved by the Ethical Committee of the Seoul St. Mary's Hospital. The patient signed informed consent.

## 2 Case presentation

**Figure 1:**
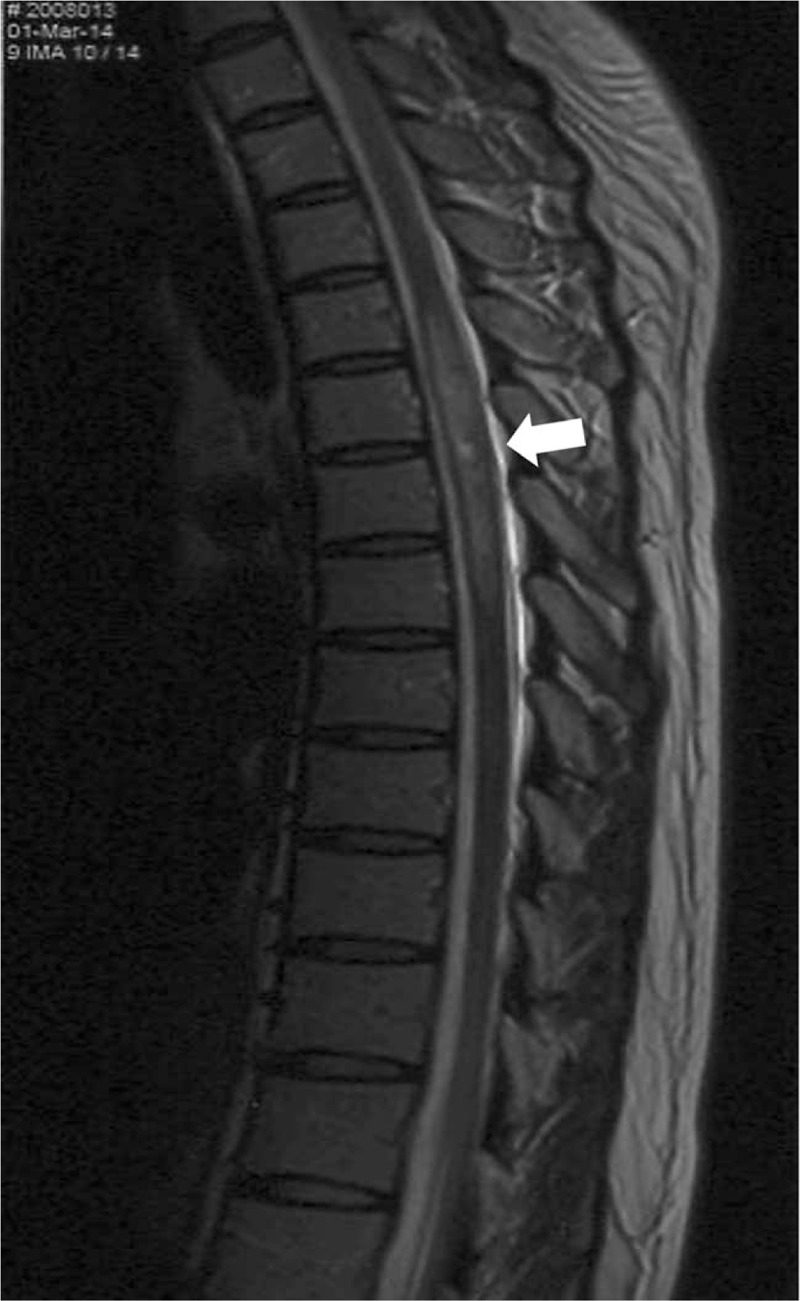
T2-weighted thoracic magnetic resonance image of the patient taken when transverse myelitis occurred. The arrow indicates high signal intensity in the spinal cord at the T5–8 level.

A 37-year-old woman was referred to a pain clinic for severe intractable pain below the T5 level. Her numerical rating scale (NRS) pain score was 10/10. She suffered from Sjögren's syndrome-induced TM 7 and 2 years before visiting the hospital, and recovered on both occasions. The first event occurred at the T5–8 level (Fig. 1), and the second at the T3–7 level. The chief complaint was gradual worsening of pain below the T5 level.

The patient's NRS pain score in the affected area was 4/10 when there was no breakthrough pain. However, the NRS score for breakthrough pain was 10/10, which was not controlled by intravenous morphine. She did not dress at home due to allodynia. The number of times she visited emergency centers to alleviate her pain increased gradually and her quality of life deteriorated. She could not walk and always used a wheelchair. She had voiding difficulties (“diaper voiding”) and feeling of incomplete bladder emptying. A neurological examination revealed paresthesia and allodynia below the T5 level. The sensory evaluation was limited by extreme pain and jerking movements. The muscle strength of both lower limbs was grade 3. The patient was maintained on 40 mg bid oxycontin, 60 μg bid fentanyl, 100 mg bid lamotrigine, 5 mg qd prednisolone, 2000 mg bid mycophenolate mofetil, and 150 mg tid pregabalin; but these were ineffective. Since it was not possible to place the patient in the supine position, spinal magnetic resonance imaging was performed under moderate sedation. Recurrence of myelitis was ruled out, as the magnetic resonance imageshowed near-complete regression of cord swelling of the upper thoracic spine. Her medical records were thoroughly reviewed to identify the reason for worsening symptoms. Tests for tuberculosis and bacterial and fungal infections were negative. A full blood count and urinalysis were normal, as was the creatinine level. The anti-aquaporin-4 IgG result was negative.

**Figure 2:**
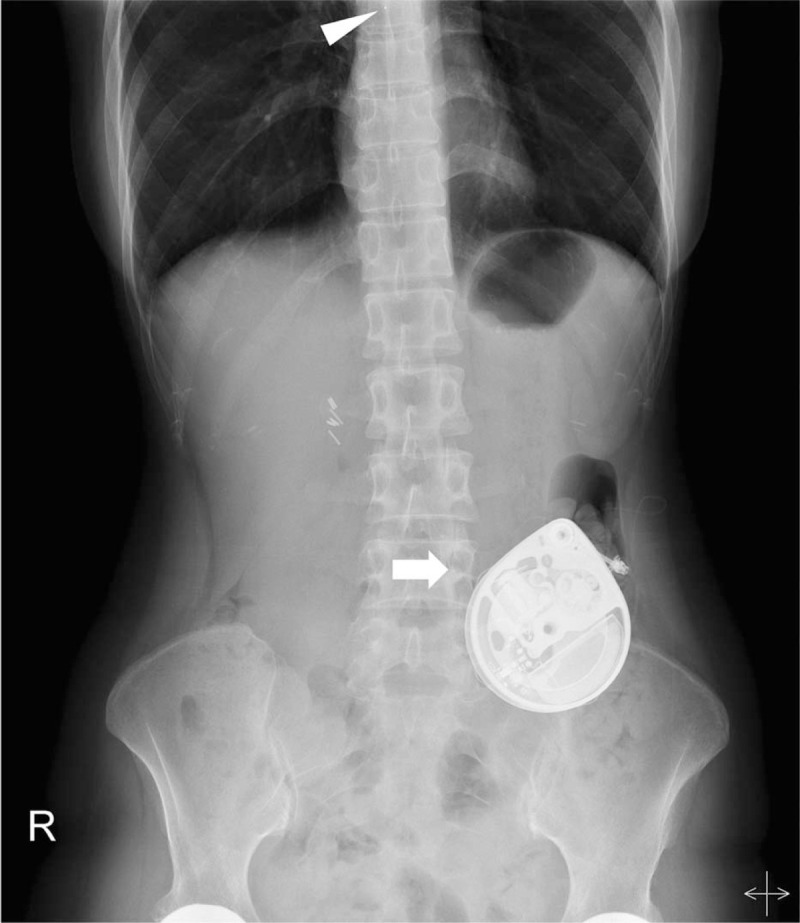
Erect abdominal radiograph taken after the procedure. The arrowhead indicates the catheter tip, and the arrow indicates the pump.

The daily morphine equivalent dose was increased due to the severity of pain, and the patient agreed to proceed with the ITP procedure. Since the patient was unable to tolerate local anesthesia of the skin, she instead received intrathecal morphine under moderate sedation. With 0.8 mg intrathecal morphine sulfate, the NRS pain score decreased from 10 to 5, with the reduction persisting for about 12 hours. She was able to wear clothes and lie straight for a limited time. Intrathecal pump (Medtronic, Minneapolis, MN, USA) was inserted into the left lower abdomen. Catheter tip was placed at the midline of the T8 level (Fig. 2). No procedural complications were encountered while inserting the pump. A continuous daily morphine dose was started at 0.9 mg/day and adjusted up to 1.4 mg/day. A bolus (myPTM; Medtronic) was added for more efficient control of breakthrough pain. A dose of 0.08 mg was delivered up to 6 times per day, and the lockout time was 1 hour. She was discharged from the hospital and could walk a few steps with assistance. The Functional Independence Measure and Barthel Index were used as disability measures.^[9,10]^ Her Functional Independence Measure score increased from 67 before implantation to 92 at the time of discharge, while her Barthel score increased from 31 to 46. After 13 months of follow-up, pain reduction had been well maintained at the NRS pain score of 5/10.

## 3 Discussion

In this case, we effectively controlled intractable neuropathic pain due to Sjögren's syndrome-related TM with an ITP. SCS is an excellent option for effective pain control in refractory pain conditions. However, SCS is more effective for spinal cord injury patients with central neuropathic pain with segmental pain near the injury site compared to those with diffuse pain below the injury site.^[11]^ Medical data of SCS on spinal cord injury groups are insufficient, and opinions are divided among experts. Additionally, in the case of patients with diffuse pain below the injury, paresthesia cannot be felt with conventional SCS. The Neuromodulation Appropriateness Consensus Committees recommend that SCS should be applied judiciously on a case-by-case basis in these patient groups.^[11]^ In this case, an ITP was selected rather than SCS, since the patient complained of severe pain in a large area below the T5 level.

To the best of our knowledge, the only case in which TM-induced neuropathic pain was controlled using an ITP was that reported by Wu et al^[12]^ There are several differences comparing our case to the Wu et al, which we will explain in turn. Their patient had a tingling sensation but no allodynia. Contrary to the case reported by Wu et al, the allodynia in our patient was so severe that it was difficult for her to get dressed.

We adjusted the ITP with a bolus option to control breakthrough pain more effectively.^[13]^ However, only different morphine doses were used during the day and at night in the Wu et al^[12]^ case. Bolash et al^[13]^ conducted a study targeting chronic pain patients, which compared the difference in oral breakthrough opioid need between a group of patients who had been assisted with basal intrathecal infusion by themselves using a wireless device with a programmed on-demand bolus, and a group of patients who had used only basal infusion. The proportion required oral opioids to control breakthrough pain was less in the group of patients who had the bolus option than in the group of patients who did not have the bolus option. The Pain Disability Index score decreased less in the group with the bolus option. The total daily intrathecal opioid intake was reduced by 34% in the group with the bolus device.

The ITP catheter tip was placed at the T8 level since the patient had pain in a large area below the T5 level. Wu et al^[12]^ did not specify the location of the ITP catheter tip. The reason we placed the tip around the T8 was to cover the affected area as much as possible. Flack et al^[14]^ implanted intrathecal infusion pumps delivering morphine in farm bred pigs, and 14 days later, the spinal cords of the animals were dissected to measure the morphine concentration. As a result, the morphine concentration decreased as a function of distance from the infusion point as it moved away from the catheter tip. Flack et al showed that the catheter tip may be critical, since the morphine distribution was very limited at that point.

Neuropathic pain is less sensitive to opioid therapy compared to other forms of chronic pain.^[16]^ There were several animal experiments that analyzed the change in spinal opioid receptor expression after nerve injury to the spinal cord of rats.^[15,16]^ The opioid receptor was downregulated in rats following nerve injury, which could explain the reason why the spinal morphine loses potency and efficacy in patients with neuropathic pain.^[15,16]^ There is high opioid demand in patients with neuropathic pain, which inevitably increases the likelihood of side effects. The main advantage of ITPs is their ability to control the dose of opioid. Moreover, there are fewer side effects than with systemic opioids, and pain can be mitigated more effectively with smaller doses. Although standard therapeutic strategies have not been established for alleviating TM-related neuropathic pain, early ITP intervention may be required when pain is uncontrolled, to reduce pain and neuropathy more quickly during subsequent TM attacks.

## Author contributions

**Conceptualization:** Yong Ho Lee, Ji Young Kim, Hyun Hwa Lee, Young Hoon Kim.

**Methodology:** Yong Ho Lee, Ji Young Kim, Hyun Hwa Lee, Young Hoon Kim.

**Supervision:** Young Hoon Kim.

**Visualization:** Ji Yeong Kim, Young Hoon Kim.

**Writing – original draft:** Ji Yeong Kim, Young Hoon Kim.

**Writing – review & editing:** Young Hoon Kim.
